# A comparative evaluation of PDQ-Evidence

**DOI:** 10.1186/s12961-018-0299-8

**Published:** 2018-03-15

**Authors:** Marit Johansen, Gabriel Rada, Sarah Rosenbaum, Elizabeth Paulsen, Nkengafac Villyen Motaze, Newton Opiyo, Charles S. Wiysonge, Yunpeng Ding, Fidele K. Mukinda, Andrew D. Oxman

**Affiliations:** 1 Global Health Cluster, Norwegian Institute of Public Health, PO Box 4404, Nydalen, N-0403 Oslo, Norway; 2 Unit for Preventive, Health Promotion and Organisation of Care, Norwegian Institute of Public Health, Oslo, Norway; 3 Centre for Informed Health Choices, Norwegian Institute of Public Health, Oslo, Norway; 4 Epistemonikos Foundation, Santiago, Chile; 5 Internal Medicine Department, Pontificia Universidad Católica de Chile, Santiago, Chile; 6 Evidence Based Health Care Program, Pontificia Universidad Católica de Chile, Santiago, Chile; 7 Division of Epidemiology and Biostatistics, Department of Global Health, Faculty of Medicine and Health Sciences, Stellenbosch University, Stellenbosch, South Africa; 8 Centre for Vaccines and Immunology, National Institute for Communicable Diseases, Johannesburg, South Africa; 9 Centre for the Development of Best Practices in Health, Yaoundé, Cameroon; 10 Cochrane Editorial Unit, Cochrane, London, United Kingdom; 11 Cochrane South Africa, South African Medical Research Council, Cape Town, South Africa; 12 Division of Epidemiology and Biostatistics, School of Public Health and Family Medicine, University of Cape Town, Cape Town, South Africa; 13 School of Public Health, University of the Western Cape, Cape Town, South Africa

**Keywords:** Health policy, Health systems, Systematic reviews, Evidence-informed health policy, Comparative study, Bibliographic databases, Clearing house, Search engine, Database searching, Search strategies, Information retrieval

## Abstract

**Background:**

A strategy for minimising the time and obstacles to accessing systematic reviews of health system evidence is to collect them in a freely available database and make them easy to find through a simple ‘Google-style’ search interface. PDQ-Evidence was developed in this way. The objective of this study was to compare PDQ-Evidence to six other databases, namely Cochrane Library, EVIPNet VHL, Google Scholar, Health Systems Evidence, PubMed and Trip.

**Methods:**

We recruited healthcare policy-makers, managers and health researchers in low-, middle- and high-income countries. Participants selected one of six pre-determined questions. They searched for a systematic review that addressed the chosen question and one question of their own in PDQ-Evidence and in two of the other six databases which they would normally have searched. We randomly allocated participants to search PDQ-Evidence first or to search the two other databases first. The primary outcomes were whether a systematic review was found and the time taken to find it. Secondary outcomes were perceived ease of use and perceived time spent searching. We asked open-ended questions about PDQ-Evidence, including likes, dislikes, challenges and suggestions for improvements.

**Results:**

A total of 89 people from 21 countries completed the study; 83 were included in the primary analyses and 6 were excluded because of data errors that could not be corrected. Most participants chose PubMed and Cochrane Library as the other two databases. Participants were more likely to find a systematic review using PDQ-Evidence than using Cochrane Library or PubMed for the pre-defined questions. For their own questions, this difference was not found. Overall, it took slightly less time to find a systematic review using PDQ-Evidence. Participants perceived that it took less time, and most participants perceived PDQ-Evidence to be slightly easier to use than the two other databases. However, there were conflicting views about the design of PDQ-Evidence.

**Conclusions:**

PDQ-Evidence is at least as efficient as other databases for finding health system evidence. However, using PDQ-Evidence is not intuitive for some people.

**Trial registration:**

The trial was prospectively registered in the ISRCTN registry 17 April 2015. Registration number: ISRCTN12742235.

**Electronic supplementary material:**

The online version of this article (10.1186/s12961-018-0299-8) contains supplementary material, which is available to authorized users.

## Background

Research evidence is essential, although insufficient for making well-informed decisions about health systems. Nevertheless, healthcare policy-makers, managers and those who support them have limited time and resources to access research when it is needed and may resort to selective use of research, such as relying on the results of primary studies rather than a more comprehensive and reliable body of evidence [1, 2].

Systematic reviews have the potential to save policy-makers and their staff the time it takes to retrieve, evaluate and synthesise results from many primary studies. Overviews of systematic reviews, where these exist, can be even more time saving, and both these document types help avoid selective use of research results. However, despite the steady increase of synthesised evidence for making well-informed decisions about health systems, many decision-makers still lack access to, awareness of, and familiarity with systematic reviews and overviews of reviews [2].

A number of existing databases and search engines provide access to systematic reviews. Cochrane Library, EVIPNet VHL, Google Scholar, Health Systems Evidence, PubMed, PDQ-Evidence and Trip, all provide access to systematic reviews that can inform healthcare decisions about how to organise, finance and govern health systems, and strategies for implementing changes. However, the comprehensiveness and ease of use differ across these resources. One strategy for minimising time and obstacles to accessing systematic reviews and overviews of reviews for policy-makers is to collect these documents in a freely available single source, and make them easy to find, through a simple ‘Google-style’ search interface. PDQ-Evidence was developed in this way to provide rapid and easy access to the best available evidence for decisions about health systems.

### PDQ-Evidence

PDQ-Evidence is intended for anyone who seeks evidence to inform health system decisions. It was launched in 2012, and is a database that facilitates rapid access to the best available evidence for decisions about health systems. PDQ-Evidence includes the four topics of delivery arrangements, financial arrangements, governance arrangements and implementation strategies. It also included evidence for decisions about public (population) health up until 2016, but no longer does. It was developed and is currently maintained by having searched the list of sources reported in Box 1, using the search strategies reported in Additional file 1, and includes five document types, namely systematic reviews, overviews of systematic reviews, structured summaries of systematic reviews, structured summaries of primary studies and primary studies (included in systematic reviews).

Box 1. PDQ-Evidence previously searched the following databases. Please see the PDQ-Evidence website for an updated list of databases currently being searched

**Table:** 

1. Cochrane Database of Systematic Reviews (CDSR)
2. Database of Abstracts of Reviews of Effectiveness (DARE)
3. Health Technology Assessment Database
4. PubMed
5. EMBASE
6. CINAHL
7. LILACS
8. PsycINFO
9. SUPPORT Summaries
10. EPPI-Centre Evidence Library
11. 3ie Systematic Reviews and Policy Briefs
12. WHO Database
13. Campbell Library
14. SURE policy briefs
15. European Observatory on Health Systems and Policies
16. DFID (United Kingdom Department for International Development) Systematic Reviews
17. NICE Public Health Guidelines and Systematic Reviews
18. Guide to Community Preventive Services
19. CADTH Rx for Change
20. McMaster Plus KT+
21. McMaster Health Forum Evidence Briefs
The detailed search strategies for PubMed, EMBASE, CINAHL, LILACS and PsycINFO can be found in the Additional file 1. All records in the other databases are screened continually by PDQ staff and volunteers, with new reviews being added to the database daily

**Fig. 1:**
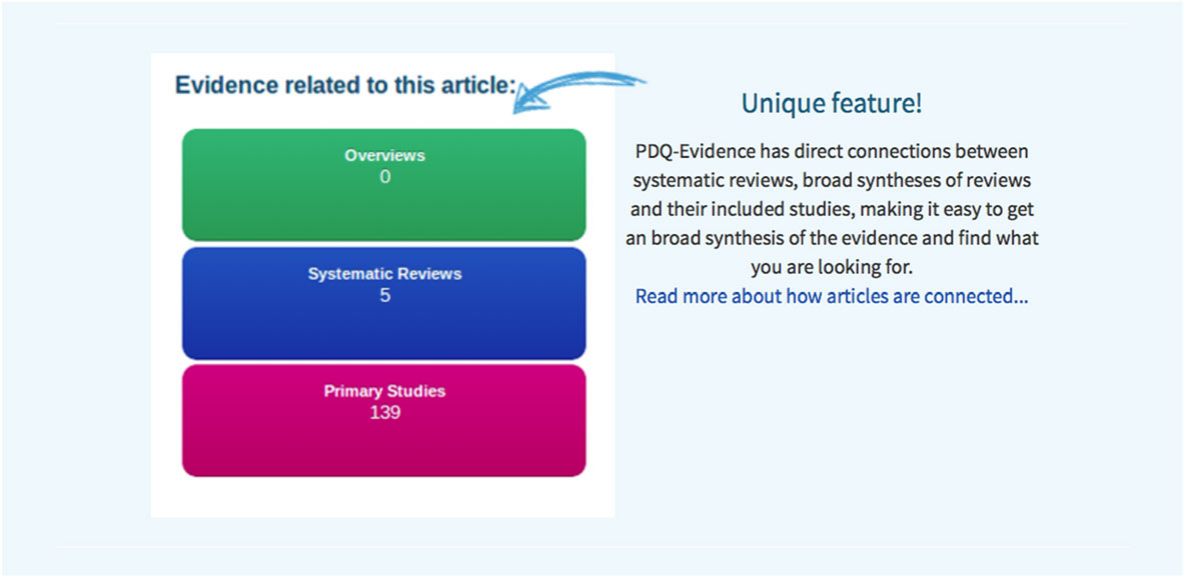
Links between systematic reviews, overviews and primary studies in PDQ-Evidence

PDQ-Evidence has the same functionalities as Epistemonikos, from which PDQ-Evidence is derived. As of Febuary 2018, PDQ-Evidence includes about 4500 records. Epistemonikos has a broader scope and includes over 200,000 systematic reviews, predominantly on clinical topics. A central and unique feature of both of these databases is the connection they provide between systematic reviews, overviews and the primary studies that are included in these reviews. While the main focus is to provide access to systematic reviews through a multilingual search, these connections provide a highly efficient method for searching across various document types that relate to the same topic, like primary studies included in reviews and reviews included in overviews. Once a relevant title is located, it is easy to move upward to more synthesised evidence, a systematic review or an overview, or to move downward to investigate each primary study that is included in a systematic review (Fig. 1).

PDQ-Evidence translates titles and abstracts of included records to facilitate searching in different languages. Languages that can currently be used for searching are English, French, Spanish and Portuguese.

For simple searches, the current design of PDQ-Evidence is modelled after a ‘Google-style’ single search field. In addition, an advanced search function enables searching in specific fields – *title*, *abstract*, *author* and *title or abstract* – and the use of Boolean logic. Both simple and advanced searches can use a two-step procedure involving the conduct of a search and using the links to connected documents (provided in the records retrieved) to access the evidence.

Results are presented as records in English. Each record includes information on document type, title, authors, journal, year, link to full text and information/links to connected documents (e.g. ‘This article includes 59 Primary studies’).

**Fig. 2:**
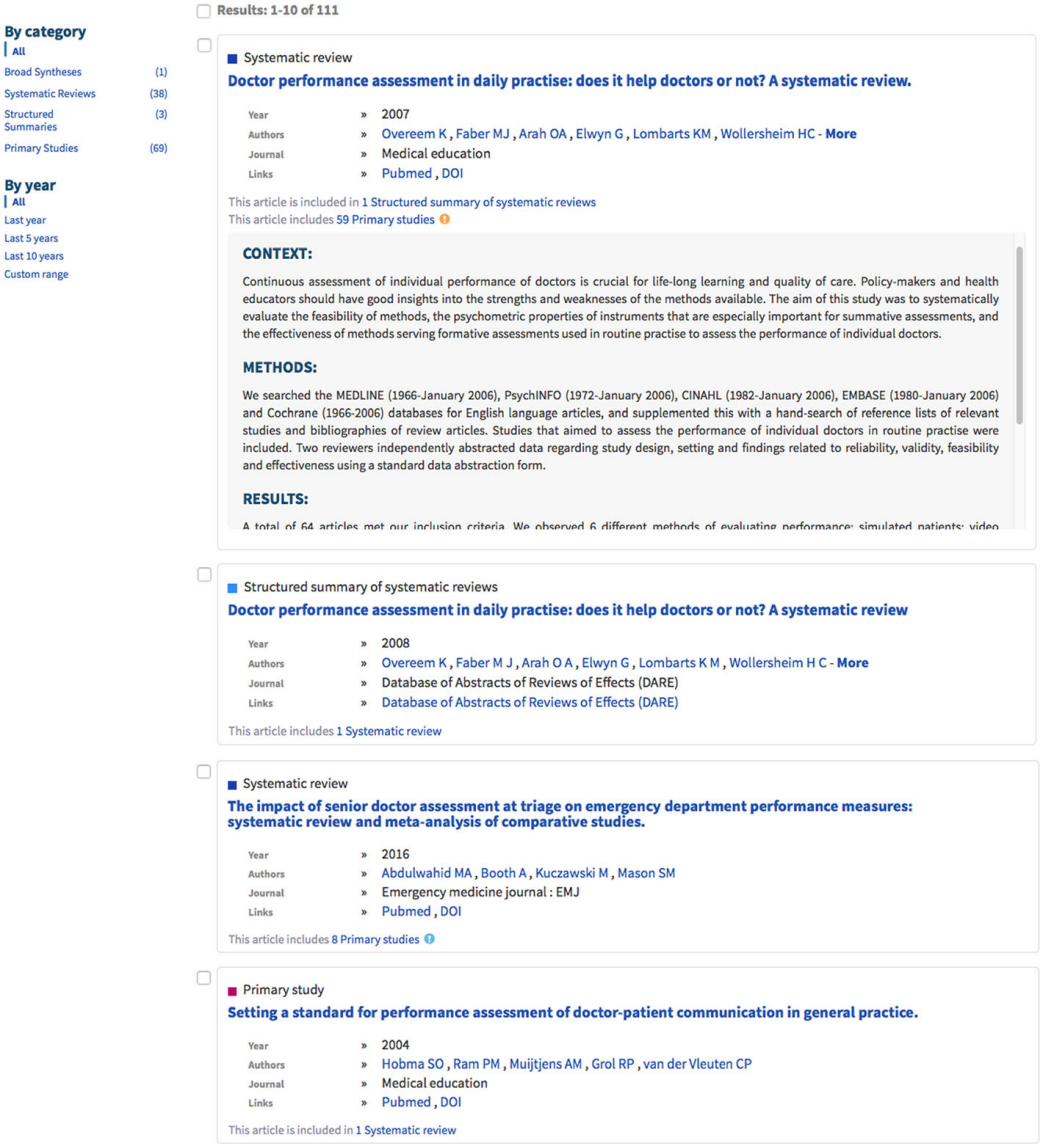
Presentation of search results in PDQ-Evidence

**Fig. 3:**
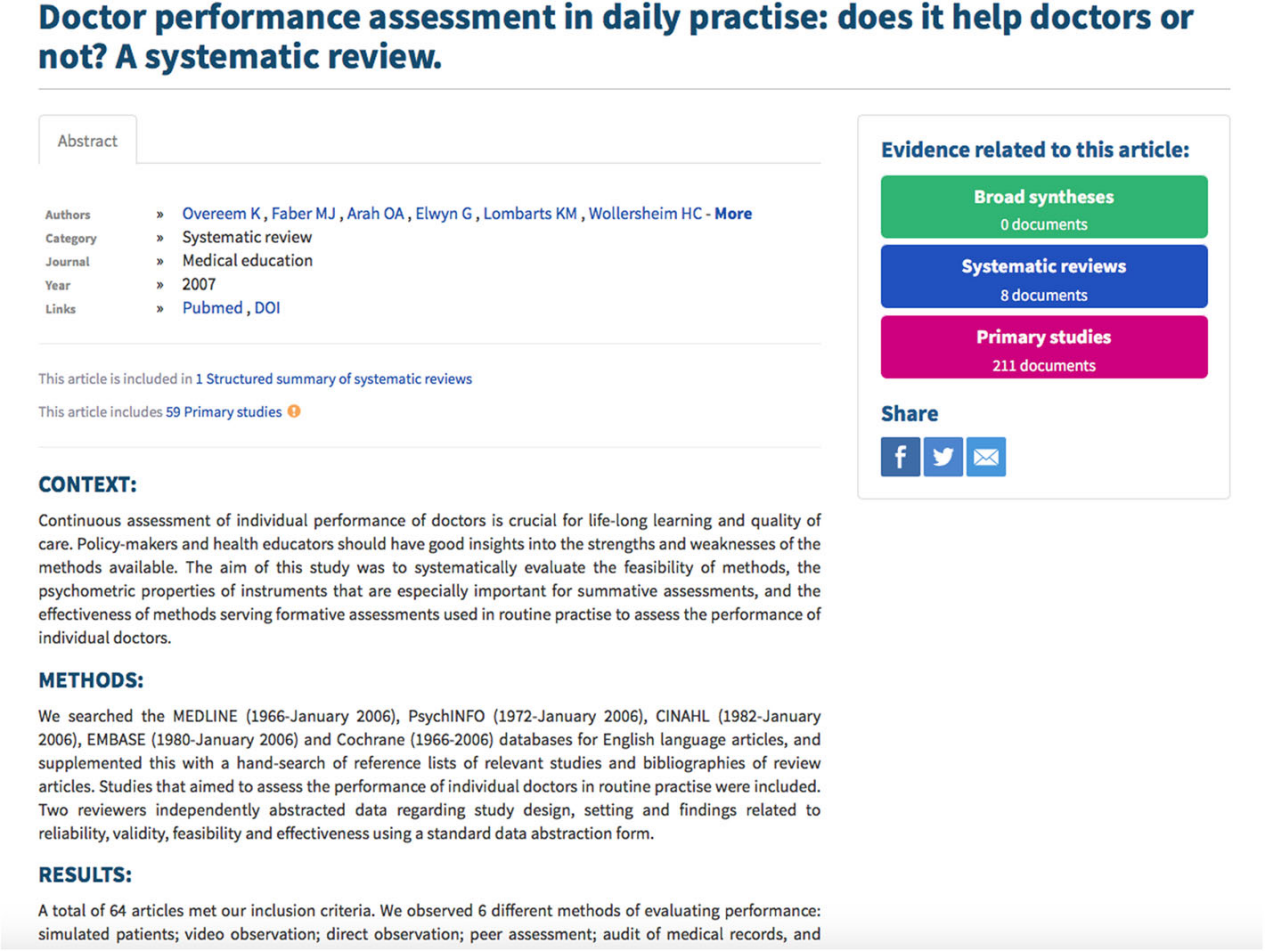
Presentation of documents in PDQ-Evidence

**Fig. 4:**
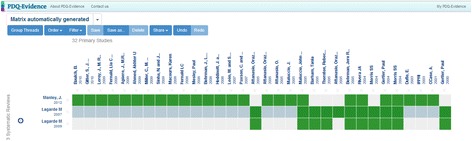
PDQ-Evidence matrix

The abstract can also be viewed directly from the results list by clicking on an arrow on the right side of a record (Fig. 2). The search results list is ordered by a relevance ranking. The list can be filtered by type of document or by year through a filter menu in the left column of the search results page. The information provided for each document (Fig. 3) consists of authors, journal, year, link to full text, information/links to connected documents (e.g. ‘This article includes 59 Primary studies’), graphic icon showing ‘Evidence related to this article’, citation reference (‘About this article’), link to export citation, and links to share on Facebook, Twitter or by email. A matrix function is currently available only to those who log in (Fig. 4). It allows the reader to view and compare included studies across two or more reviews.

PDQ-Evidence is a work in progress, with planned improvements including further work on translations, save searches, automatic updates, print and email search results or abstracts, browsing functions, filtering by country or groups of countries, further developments of the matrix functionality and notifications of new evidence. Being a work in progress, PDQ-Evidence may, at the time of publication, differ from how we have described it in this evaluation.

The following people have contributed to the development of PDQ-Evidence:

- Gabriel Rada, Evidence-Based Health Care Program, Pontificia Universidad Católica de Chile. Director of Epistemonikos Project
- Daniel Pérez, CTO Epistemonikos and other members of the Epistemonikos team
- Susan Munabi Babigumira, Simon Lewin, Jenny Moberg, Andy Oxman, Sarah Rosenbaum, Global Health Cluster Norwegian Institute of Public Health
- Shaun Treweek, Health Services Research Unit, University of Aberdeen, Scotland

Most of the technical developments rely on the software and collaborators of the Epistemonikos project [3]. Initial user testing of PDQ-Evidence was performed prior to this evaluation of the database and focused on identifying aspects of PDQ-Evidence’s design, functionality and content that needed adjustment, as well as generating new ideas for further improvement. In this article, we report an evaluation of how well the PDQ-Evidence database performs compared to other databases when searching for systematic reviews.

## Methods

### Participants

We recruited a convenience sample of participants. Members of the Supporting Use of Research Evidence (SURE) collaboration, the Evidence-Informed Health Policy Network (EVIPNet), and the Cochrane review group Effective Practice and Organisation of Care (EPOC) identified and recruited healthcare policy-makers, managers and health researchers to evaluate the PDQ-Evidence database.

### Searching for systematic reviews

To evaluate PDQ-Evidence, participants searched for systematic reviews in PDQ-Evidence and we compared its performance to searches for systematic reviews in the three databases and three search engines (all referred to as databases in this article) described below.

#### Cochrane Library

Cochrane Library is a collection of seven databases where six of the seven contain different types of high-quality, independent evidence to inform healthcare decision-making.

#### EVIPNet VHL

The EVIPNet Virtual Health Library supports country teams in evidence-informed health policy-making and provides evidence and tools for the activities of knowledge translation platforms, particularly in low- and middle-income countries.

#### Google Scholar

Google Scholar provides a simple way to broadly search for scholarly literature.

#### Health Systems Evidence

Health Systems Evidence is a continuously updated repository of syntheses of research evidence about governance, financial and delivery arrangements within health systems, and about implementation strategies that can support change in health systems.

#### PubMed

PubMed comprises more than 28 million citations for biomedical literature from MEDLINE, life science journals and online books.

#### Trip

Trip is a clinical search engine designed to allow users to quickly and easily find and use high-quality research evidence to support their practice or care.

All participants were asked to search three databases – PDQ-Evidence and two of the six databases listed above in which they would normally have searched or which they believe would be most likely to enable them to quickly find a relevant review. To avoid ‘learning bias’ we randomised the search order of the databases. Either PDQ-Evidence was searched first and then the two self-selected databases in any order as the second and third database, or PDQ-Evidence was searched last after the two selected databases in any order as the first and second database.

All participants were asked to try to find an answer to two health system questions in all three databases. Participants were asked to search for one predefined question and one question of their choice (Additional file 2). To obtain a fair comparison between PDQ-Evidence and any of the other databases that participants could choose from, we limited the database selection to databases that include relevant systematic reviews that address the predefined question. Participants were asked to spend no more than 10 minutes on each search, or a total of approximately 1 hour to do two searches in each of three databases.

### Outcomes

Each participant was asked to record the following for each database searched:

#### Primary outcomes

1. Was a systematic review that addresses the question found (Yes/No)? For the comparison databases (the two databases selected by the participants) this outcome was defined as: “Was a systematic review that addresses the question found in either of the two databases?”
2. Time taken to find a systematic review that addresses the question

#### Secondary outcomes (asked regarding each database)

3. Assessments of the databases with four response options:
  - Ease of use (from very difficult to very easy)
  - Time spent on searching (from much too much time to very little time)

Additional outcomes (open ended questions, only asked regarding PDQ-Evidence)

4. Likes
5. Dislikes
6. Challenges
7. Suggestions for improvements

We used SurveyGizmo to collect data. A printed version of the questionnaire that we used can be found in Additional file 3.

### Analysis

All quantitative analyses of the primary outcomes were conducted separately for the predefined question and the question chosen by the participants themselves. Data for the first outcome (whether a review was found) were analysed using McNemar’s test for agreement between paired frequencies by comparing the proportion of searches that yield a relevant answer to the question. Data for the second outcome (time taken to find a review) were analysed using Generalised Estimation Equations allowing for repeated observations (three, one for each database) for each participant; the link-function was the identity (normal distribution) and the database used was an independent variable (PDQ-Evidence was the reference to which the other databases were compared) in the model. The main analyses included only participants that found a systematic review that answered the question. Sensitivity analyses including all participants were performed, where the time was set to 10 minutes for all databases if a relevant systematic review was not found.

We compiled all the comments received in response to the secondary outcomes and prepared a table summarising these.

### Sample size

We calculated the sample size based on McNemar’s test for agreement using Miettinen’s approximation to the normal distribution. We wanted to be able to detect a difference compared to finding the answer to the predefined question in PDQ-Evidence of at least 20 percentage points, with a power of 80%, and α = 0.05. We assumed a 50/50 chance of participants finding the answer to a predefined question in at least one of the databases they selected. We also assumed that the binary correlation of the results from the sources searched (PDQ-Evidence and the two self-selected databases) was 0 (worst case scenario, as a negative correlation is unlikely and a positive correlation would yield a lower sample size).

Based on these assumptions, we estimated that we would need 94 participants.

## Results

We recruited participants between 16 October and 6 February 2015. A total of 89 people completed the study (Table 1) and 83 were included in the primary analyses. Six participants who completed the study were excluded from the primary analyses because of errors in their data that could not be corrected.

**Table 1:** Characteristics of participants

	PDQ-Evidence first	PDQ-Evidence last	Total
	*N* = 39	*N* = 50	*N* = 89
Countries	Cameroon 3, Canada 2, Chile 1, China 1, India 1, Iran 2, Italy 2, Kenya 2, Lebanon 2, Nigeria 1, Norway 4, Senegal 1, South Africa 5, Switzerland 1, United Kingdom 4, United States 3, missing 4	Argentina 1, Brazil 1, Canada 1, Italy 3, Japan 1, Kenya 6, Lebanon 4, Nigeria 1, Norway 7, Pakistan 1, South Africa 5, Sweden 1, Switzerland 4, United Kingdom 2, United States 4, missing 8	Argentina 1, Cameroon 3, Brazil 1, Canada 3, Chile 1, China 1, India 1, Iran 2, Italy 5, Japan 1, Kenya 8, Lebanon 6, Nigeria 2, Norway 11, Pakistan 1, Senegal 1, South Africa 10, Sweden 1, Switzerland 5, United Kingdom 6, United States 7, missing 12
Training or work experience^a^			
Health policy-maker	9 (23%)	10 (20%)	19 (21%)
Health systems planning or analysis	8 (21%)	10 (20%)	18 (20%)
Healthcare professional	22 (56%)	26 (52%)	48 (54%)
Management of health services	8 (21%)	11 (22%)	19 (21%)
Research	31 (79%)	41 (82%)	72 (81%)
Current position^a^			
Health professional	5 (13%)	2 (4%)	7 (8%)
Health technology assessment consultant	0	1 (2%)	1 (1%)
Librarian	1 (3%)	1 (2%)	2 (2%)
Manager	4 (10%)	4 (10%)	8 (9%)
MSc or PhD student	2 (5%)	1 (2%)	3 (3%)
Policy-maker	4 (10%)	1 (2%)	5 (6%)
Professor or teacher	5 (13%)	2 (4%)	7 (8%)
Researcher	32 (82%)	40 (80%)	72 (81%)
Technical support staff	5 (13%)	6 (12%)	11 (12%)
First language			
English	18 (46%)	23 (46%)	41 (46%)
Other	21 (54%)	27 (54%)	48 (54%)
Frequency of searches for systematic reviews			
I never search myself	2 (5%)	2 (4%)	4 (4%)
Less than once a year	2 (5%)	0	2 (2%)
Once or twice a year	7 (18%)	18 36(%)	25 (28%)
Once or twice a month	13 (33%)	19 (38%)	32 (36%)
Once or twice a week	13 (33%)	10 (20%)	23 (26%)
Many times a week	2 (5%)	1 (2%)	3 (3%)
Places normally searched for health system questions^a^			
Cochrane Library	25 (62%)	37 (74%)	62 (70%)
EVIPNet	0	0	0
Google Scholar	11 (28%)	18 (36%)	29 (33%)
Health Systems Evidence	1 (3%)	2 (4%)	3 (3%)
PDQ-Evidence	2 (5%)	2 (4%)	4 (4%)
PubMed^b^	32 (82%)	39 (78%)	71 (80%)
TRIP	3 (8%)	3 (6%)	6 (7%)

^a^Participants could give more than one response

^b^Includes five responses that were MEDLINE

Participants came from 21 countries, namely Argentina, Brazil, Cameroon, Canada, Chile, China, India, Iran, Italy, Japan, Kenya, Lebanon, Nigeria, Norway, Pakistan, Senegal, South Africa, Sweden, Switzerland, United Kingdom and United States (Table 1). Less than half (46%) had English as their first language. They currently worked as health professionals, health technology assessment consultants, librarians, managers, MSc or PhD students, policy-makers, professors or teachers, researchers, or technical support staff. They had training or work experience from health policy-making, health systems planning or analysis, management of health services, or as healthcare professionals or researchers. Only four participants had not previously searched for a systematic review; 26% searched for a systematic review either once or twice a week, 36% once or twice a month, and 28% once or twice a year. The sources they normally used to find systematic reviews were PubMed (80%), Cochrane Library (70%) and Google Scholar (33%).

All 83 participants included in the analysis searched PDQ-Evidence for both the pre-defined questions and their own questions. Most participants chose PubMed (65, 78%) and Cochrane Library (57, 69%) as the other two databases that they searched (Table 2).

**Table 2:** Databases searched by the participants and the yield of each database search

Database	N (%)^a^	Pre-defined questions	Own questions
		Found a review	Found a review
		N (%)^b^	N (%)^b^
PDQ-Evidence	83 (100%)	72 (87%)	48 (58%)
PubMed	65 (78%)	37 (57%)	37 (57%)
Cochrane Library	57 (69%)	37 (65%)	31 (54%)
Google Scholar	21 (25%)	15 (71%)	14 (67%)
Health Systems Evidence	10 (12%)	7 (70%)	5 (50%)
Trip	10 (12%)	7 (70%)	7 (70%)
EVIPNet	3 (4%)	2 (67%)	1 (33%)

^a^Proportion of 83 participants who searched each database

^b^Proportion of participants who searched each database that found a systematic review addressing the question

Participants were more likely to find a systematic review using PDQ-Evidence than using Cochrane Library or PubMed for the pre-defined questions (87%, 57% and 65% for PDQ-Evidence, PubMed and Cochrane Library, respectively). For their own questions, the proportion of participants who found a systematic review was similar for the three databases (58%, 57% and 54%, respectively).

### Finding a review

Overall, when comparing PDQ-Evidence to searches using two other databases (combined), participants were more likely to find a systematic review using PDQ-Evidence when searching for an answer to a predefined question (Table 3), and less likely using PDQ-Evidence when searching for an answer to their own questions. However, there was an order effect when searching for an answer to their own questions. Participants were more likely to find a review when PDQ-Evidence was searched first than when it was searched last (69% first vs. 49%) (Table 4).

**Table 3:** Participants who found a systematic review for predefined questions

Group			Found review searching the other two databases		Total
			No	Yes	
PDQ-Evidence first	Found review searching PDQ-Evidence	No	2 (5.6%)	1 (2.8%)	**3 (8.3%)**
		Yes	9 (25.0%)	24 (66.7%)	**33 (91.7%)**
	Total		**11 (30.6%)**	**25 (69.4%)**	36 (100.0%)
PDQ-Evidence last	Found review searching PDQ-Evidence	No	4 (8.5%)	4 (8.5%)	**8 (17.0%)**
		Yes	6 (12.8%)	33 (70.2%)	**39 (83.0%)**
	Total		**10 (21.3%)**	**37 (78.7%)**	47 (100.0%)
Total	Found review searching PDQ-Evidence	No	6 (7.2%)	5 (6.0%)	**11 (13.3%)**
		Yes	15 (18.1%)	57 (68.7%)	**72 (86.7%)**
	Total		**21 (25.3%)**	**62 (74.7%)**	83 (100.0%)

**Table 4:** Participants who found a systematic review for their own questions

Group			Found review searching the other two databases		Total
			No	Yes	
PDQ-Evidence first	Found review searching PDQ-Evidence	No	7 (19.4%)	4 (11.1%)	**11 (30.6%)**
		Yes	5 (13.9%)	20 (55.6%)	**25 (69.4%)**
	Total		**12 (33.3%)**	**24 (66.7%)**	36 (100.0%)
PDQ-Evidence last	Found review searching PDQ-Evidence	No	10 (21.3%)	14 (29.8%)	**24 (51.1%)**
		Yes	2 (4.3%)	21 (44.7%)	**23 (48.9%)**
	Total		**12 (25.5%)**	**35 (74.5%)**	47 (100.0%)
Total	Found review searching PDQ-Evidence	No	17 (20.5%)	18 (21.7%)	**35 (41.6%)**
		Yes	7 (8.4%)	41 (49.4%)	**48 (57.8%)**
	Total		**24 (28.9%)**	**59 (71.1%)**	83 (100.0%)

When searching for a systematic review for a predefined question, 72 (87%) participants found a systematic review using PDQ-Evidence compared to 62 (75%) participants using the two other databases they chose to search (12% more with PDQ-Evidence than with two databases; 95% CI 2% to 23%) (Table 3). For participants who searched PDQ-Evidence first, 22% more (95% CI 5% to 39%) found a systematic review using PDQ-Evidence (92%) compared to the other two databases (69%). When PDQ-Evidence was searched last, there was little if any difference – 83% for PDQ-Evidence compared to 79% for the other two databases (4% more; 95% CI 19% fewer to 28% more).

When searching for a systematic review for their own question, 48 (58%) participants found a systematic review using PDQ-Evidence compared to 59 (71%) participants using the two other databases they chose to search (13% fewer with PDQ-Evidence than with the other two databases; 95% CI 25% to 1% fewer) (Table 4). For participants who searched PDQ-Evidence first, 3% more (95% CI 14% fewer to 19% more) found a systematic review using PDQ-Evidence (69%) compared to the other two databases (67%), whereas, when PDQ-Evidence was searched last, 26% fewer (95% CI 47% to 4% fewer) found a systematic review with PDQ-Evidence (49%) compared to the other two databases (75%).

Overall, 65 of the 83 (78%) participants chose to search PubMed, 57 (69%) chose to search Cochrane Library and 21 or fewer (less than 25%) of the participants chose to search any of the other four databases (Table 2); therefore, the estimates of how long it took to find a systematic review for those databases are not robust. There were not large differences in the average times it took participants to find a systematic review using PDQ-Evidence, PubMed or Cochrane Library.

Among the participants that found a systematic review that addressed the pre-defined questions, the mean time taken to find a review was 5.3, 5.1 and 6.1 minutes for PDQ-Evidence, PubMed and Cochrane Library, respectively (Table 5). Relative to PDQ-Evidence, there was little or no difference in how long it took to search PubMed (0.1 minutes less; 95% CI 1.7 minutes less to 1.4 minutes more) or Cochrane Library (0.8 minutes more; 95% CI 0.7 minutes less to 2.4 minutes more).

**Table 5:** Time taken to find a systematic review for participants who found a review for pre-defined questions

		Time in minutes			
Database	N	Mean	Standard deviation	Minimum	Maximum
PDQ-Evidence	72	5.25	3.87	1	19
PubMed	37	5.11	3.26	1	14
Cochrane Library	37	6.08	2.49	1	10
Google Scholar	15	4.93	2.91	1	10
Health Systems Evidence	7	5.29	2.87	2	10
Trip	7	8.14	11.5	1	34
EVIPNet	2	9	0	9	9

When all the participants that searched each database were included, assuming 10 minutes for each participant who did not find a systematic review for the pre-defined question, the mean time taken to find a review was 5.2, 6.5 and 5.8 minutes for PDQ-Evidence, PubMed and Cochrane Library, respectively (Table 6), corresponding to 1.2 minutes more for PubMed (95% CI 0.5 to 3.0 minutes more) and 0.6 minutes more for Cochrane Library (95% CI 1.2 minutes less to 2.3 minutes more).

**Table 6:** Time taken to find a systematic review for all participants for pre-defined questions^a^

		Time in minutes			
Database	N	Mean	Standard deviation	Minimum	Maximum
PDQ-Evidence	83	5.24	3.83	1	19
PubMed	64	6.48	7.53	1	60
Cochrane Library	57	5.79	2.66	1	12
Google Scholar	21	5.81	3.04	1	10
Health Systems Evidence	10	8.2	6.65	2	25
Trip	10	6.90	9.68	1	34
EVIPNet	3	7	3.46	3	9

^a^Assuming 10 minutes for participants who did not find a systematic review

Among the participants that found a systematic review that addressed their own questions, the mean time taken to find a review was 4.7, 5.4 and 4.7 minutes for PDQ-Evidence, PubMed and Cochrane Library, respectively (Table 7). This suggests that, on average, there was also little if any difference in the time it took to find a systematic review using either PubMed or Cochrane Library compared to PDQ-Evidence (0.7 minutes more for PubMed; 95% CI 0.7 minutes less to 2.2 minutes more).

**Table 7:** Time taken to find a systematic review for participants who found a review for their own questions

		Time in minutes			
Database	N	Mean	SD	Minimum	Maximum
PDQ-Evidence	48	4.69	3.18	1	14
PubMed	36	5.42	3.53	1	15
Cochrane Library	31	4.74	3.19	1	10
Google Scholar	14	3.64	3.15	0	11
Health Systems Evidence	5	5	5.39	1	14
Trip	6	3.83	1.72	2	6
EVIPNet	1	10	0	10	10

When all the participants that searched each database were included, assuming 10 minutes for each participant who did not find a systematic review for their own question, the mean time taken to find a review was 5.1, 5.3 and 4.9 minutes for PDQ-Evidence, PubMed and Cochrane Library, respectively (Table 8). This suggests again that there was little or no difference in the average time it took to find a systematic review using either PubMed or Cochrane Library compared to PDQ-Evidence (0.2 minutes more for PubMed; 95% CI 1.0 minutes less to 1.4 minutes more).

**Table 8:** Time taken to find a systematic review for all participants for their own questions^a^

		Time in minutes			
Database	N	Mean	SD	Minimum	Maximum
PDQ-Evidence	83	5.07	3.97	0	20
PubMed	64	5.28	3.29	1	15
Cochrane Library	57	4.89	3.29	1	11
Google Scholar	21	4.57	3.63	0	11
Health Systems Evidence	10	4.50	3.89	2	10
Trip	9	4.44	1.94	2	8
EVIPNet	3	6.00	3.46	4	10

^a^Assuming 10 minutes for participants who did not find a systematic review

### Perceived ease of use

As noted above, 21 or fewer of the participants (less than 25%) chose to search four of the databases. Therefore, the estimates of the perceived ease of use are not robust (Table 9). On a four-point scale (from 1 = very easy to 4 = very difficult), the 83 participants that searched PDQ-Evidence rated the ease of use to be between very easy and easy (mean 1.65).

**Table 9:** Perceived ease of use^a^

Database	N	Mean	Standard deviation	Minimum	Maximum
PDQ-Evidence	83	1.65	0.63	1	3
PubMed	65	2.18	0.81	1	4
Cochrane Library	57	2.04	0.63	1	3
Google Scholar	21	1.62	0.59	1	3
Health Systems Evidence	10	2.40	0.97	1	4
Trip	10	1.50	0.71	1	3
EVIPNet	3	3	1	22	4

^a^Measured on a four-point scale: from 1 = very easy to 4 = very difficult

The 65 participants that searched PubMed rated the ease of use to be between easy and difficult, (mean 2.18). This indicates that, on average, they found PubMed to be slightly more difficult to use than PDQ-Evidence (difference 0.53 points more difficult, 95% CI 0.31 to 0.76).

The 57 participants that searched Cochrane Library rated the ease of use to be between easy and difficult (mean 2.04), indicating that, on average, they also found Cochrane Library to be slightly more difficult to use than PDQ-Evidence (difference 0.40 points more difficult, 95% CI 0.15 to 0.62).

Participants rated how much time they spent searching each database on a four-point scale (from 1 = very little time to 4 = much too much time). The 83 participants that searched PDQ-Evidence rated the time spent searching to be between very little time and not too much time (mean 1.5) (Table 10).

**Table 10:** Perceived time spent searching^a^

Database	N	Mean	SD	Minimum	Maximum
PDQ-Evidence	83	1.51	0.67	1	4
PubMed	65	2.17	0.84	1	4
Cochrane Library	57	1.91	0.63	1	3
Google Scholar	21	2.00	1.00	1	4
Health Systems Evidence	10	2.20	1.03	1	4
Trip	10	1.70	0.67	1	4
EVIPNet	3	2.00	1.00	1	3

^a^Measured on a four-point scale: from 1 = very little time to 4 = much too much time

The 65 participants that searched PubMed rated the time spent between not too much time and too much time (mean 2.2), indicating that, on average, they perceived PubMed to take slightly more time to search than PDQ-Evidence (0.7 points more; 95% CI 0.4 to 0.9 more).

The 57 participants that searched Cochrane Library rated the time spent searching to be between very little time and not too much time (mean 1.9), indicating that, on average, they also found Cochrane Library to take slightly more time to search than PDQ-Evidence (difference 0.4 points more, 95% CI 0.2 to 0.7 more).

### Feedback regarding PDQ-Evidence

We asked the 89 participants four open-ended questions about PDQ-Evidence regarding what they liked, what they disliked, challenges and suggestions for improvements (Table 11). Overall, the feedback was more positive than negative. We grouped the feedback for each of these questions into four main categories, namely structure, content, searching and records. The structure category includes the database layout, the use of colour codes to distinguish between different types of publications (systematic reviews, overviews of reviews and primary studies), and the linking of studies to related evidence. The content category includes information about PDQ-Evidence, the comprehensiveness of the database, topics and publication types covered, access to full text, how up-to-date the database is, and issues regarding the quality assessment of its content. The searching category includes search and navigation functionalities (including indexing of content and the possibility to search in languages other than English), the possibility to filter the database content, the geographical distribution of studies, help functions, issues regarding the search history, and the perceived ease of use and speed. The records category includes how records are displayed, the content of individual records, issues related to saving end exporting records, issues regarding the relevance of search results, and the number of records retrieved (too few or too many).

**Table 11:** Additional outcomes

PDQ-Evidence: Additional outcomes																											
Feedback			STRUCTURE			CONTENT							SEARCHING							RECORDS							
Outcomes	Number that gave feedback out of 89 asked	Number of comments	Interface/layout	Colour codes	Links to related documents	Information about PDQ	Comprehensiveness	Topics	Publication types	Full text	Year/up-to-date	Quality assessment	Search functionality	Categories/filters	Geographical distribution	Search help	Search history	Ease of use	Speed	Record display/format	Record content	Save and export	Relevance	Number of hits (too few/many)	General	Other	No comment
Likes	89	153	24	6	23	0	0	2	7	0	1	0	9	21	2	0	2	18	9	8	3	1	8	1	1	7	0
Dislikes	58	64	2	1	1	1	0	3	4	3	4	0	11	5	0	1	0	4	1	4	0	1	6	7	0	5	31
Challenges	54	63	4	0	2	1	5	1	3	4	1	1	8	1	0	1	1	8	0	2	0	0	3	6	1	10	35
Suggestions	61	77	5	0	2	1	6	0	2	5	0	0	14	8	0	9	1	2	0	3	5	4	2	0	4	4	28

#### Likes

All 89 participants gave feedback on what they liked about PDQ-Evidence. Of the 153 individual comments, we excluded eight, categorised as ‘general’ or ‘other’. These comments were too general, did not refer to PDQ-Evidence, referred to the survey itself, gave too little information, or the feedback was unclear.

Positive feedback referred mainly to two categories, structure and searching. Typical comments included:

“Clear uncluttered screen…”

“The best is that we can find the included primary studies from this database”

“The filter search results presentation on the left is clear and practical”

“It is straight forward”

Participants also liked the use of colour codes, the variety of topics covered, the inclusion of publication types, such as systematic reviews and structured summaries, with links to related evidence (including primary studies), and that the summaries seemed to be up-to-date. In addition, participants liked the search functionality, the geographic distribution of records, the display of the search history, the quick response when searching PDQ-Evidence, how PDQ-Evidence displays search results and records, the possibility to export records, that records seem to be relevant, and the not overwhelming number of search results.

#### Dislikes

Fifty-eight participants gave feedback on what they did not like about PDQ-Evidence. Of the 64 individual comments, we excluded five, categorised as ‘general’ or ‘other’. These comments gave too little information, the feedback was unclear, or referred to technical issues not related to PDQ-Evidence.

Negative feedback referred mainly to the searching category. Typical comments included:

“If you cannot put all combinations in one sentence it is too cumbersome, a red plus is easy to mix up with the blue one – better with a minus …”

“Not sensitive to spelling variations”

“I would prefer to use MESH terms”

Some participants also did not find the layout very user friendly and disliked the colours used for colour codes. Although some participants appreciated the linking to related evidence, others found this way of structuring the database confusing:

“It could be easy to get lost when you start ‘navigation’ through the net of evidence (where I started?? Where I am now??)”

Some participants could not find information on the database content. Others disliked that PDQ-Evidence excluded clinical topics and would have liked to find additional primary studies, not only those included in PDQ-Evidence’s systematic reviews. Some were disappointed they could not find full text articles, were concerned the database was not up-to-date, found the filter option unsatisfactory, and found the search help unclear. Others felt that PDQ-Evidence was not intuitive and easy to use, and that it was time consuming to search. Some were unhappy with how PDQ-Evidence displayed the search results, and others could not find how to save records to file. Two comments on the relevance of records were:

“I did find more relevant publications in PubMed …”

“I was surprised my initial ‘financial incentives breast feeding’ search found 0 articles when there were clearly articles there.”

Comments on the number of hits retrieved or displayed varied from too few to too many hits.

#### Challenges

Fifty-four participants gave feedback on challenges related to PDQ-Evidence. Of the 63 individual comments, we excluded 11, categorised as ‘general’ or ‘other’. These comments were too general, referred to the survey itself, gave too little information, the feedback was unclear, or referred to technical issues not related to PDQ-Evidence. However, one of the general, but legitimate comments worth mentioning, questioned the existence of PDQ-Evidence, asking “Why yet another database?”

Some participants referred to challenges they ran into during the evaluation, others to challenges for PDQ-Evidence’s developers and providers. Comments referred to a not so user-friendly layout, confusing links to a large amount of related evidence and unclear information about the database content. PDQ-Evidence does not seem to be comprehensive, which was found challenging, as additional databases need to be searched. Knowing which topics and publication types are covered is a challenge, and so is the lack of full text. Keeping the database up to date, and making sure the content is quality assessed was perceived as being challenging for the database developers. Regarding the search functionality, one of the participants had the following comment:

“To compete against existing search engines, a better and more comprehensive system is required.”

Other comments mentioned the need to provide better filter option techniques, the lack of specific guidance on how to search and problems with navigating back to the search history. Comments on the ease of use that suggest searching PDQ-Evidence was not intuitive to some first-time users included:

“New to me; need to get used to it.”

“Had never used this database before, so it was challenging, but I guess with more frequent use it will get better.”

Other challenges included the fact that only a few records were displayed at a time thus ‘hiding’ the additional records found, that many irrelevant records were retrieved, and that there was a huge number or a paucity of hits.

#### Suggestions

Sixty-one participants had suggestions for how to improve PDQ-Evidence. Of the 77 individual comments, we excluded eight, categorised as ‘general’ or ‘other’. These suggestions were too general, gave too little information, the feedback was unclear, or it referred to technical issues not related to PDQ-Evidence.

Participants suggested improvements to the layout. For example, it should be more colourful and use bigger fonts. It was suggested that linking to related evidence should be less extensive, the information about PDQ-Evidence should give clear examples of evidence included, the database should include additional resources to make it more comprehensive, the inclusion criteria for publication types should be broader, full-text publications should be made available, the search functionality should preferably include predefined keywords or MeSH terms, an extended filter option would be an improvement, sorting hits based on relevancy, and a PICO-format search option should be included.

Participants had specific suggestions for how to improve the search help, including:

“… a ‘route map’ option when you get lost.”

“A three minute, once-off orientation video.”

“… some prompts or auto-suggestion in search screen, or an ‘online chat assistance’ in return for membership or a fee.”

It was also suggested that PDQ-Evidence should be easier to use, it should be possible to display more than 10 records at a time, abstracts should be available in different languages and include author contact details, it should be possible to export records easily, and records retrieved should be relevant to the search question.

## Discussion

Participants were more likely to find a systematic review using PDQ-Evidence than using any of the other databases to which it was compared. When searching for an answer to a predefined question, they were more likely to find a review using PDQ-Evidence than using any two other databases. However, when searching for an answer to their own questions, they were less likely to find a systematic review when compared to using any two other databases. A possible explanation for this difference is that some of the questions that participants asked were not within the scope of PDQ-Evidence, whereas all the pre-defined questions were within the scope of all seven databases. In addition, the order in which the databases were searched might have affected the results since participants were more likely to find a systematic review when PDQ-Evidence was searched first than when it was searched last. This finding is difficult to explain; yet, given the small numbers, it is possible that it is a spurious finding.

Overall, it took slightly less time to find a systematic review using PDQ-Evidence. Participants perceived that it took less time, and most participants perceived PDQ-Evidence to be slightly easier to use than the two other databases that were searched. There were conflicting views about the design of PDQ-Evidence.

Participants selected databases with which they were familiar. Few listed PDQ-Evidence as a database they normally would use, and our primary analyses compared PDQ-Evidence alone to two other self-selected databases. Thus, the design of the study favoured the databases to which PDQ-Evidence was compared.

Strengths of this study include diverse participants from low-, middle-, and high-income countries, direct comparisons with other databases using both predefined and their own questions, and allowing participants to select the comparison databases based on what they would normally have searched or what they believed would be most likely to enable them to quickly find a relevant review.

Weaknesses of this study include that we had difficulty recruiting participants and did not achieve the estimated required sample size. In addition, few participants selected four of the databases, so that it was not possible to draw reliable conclusions about PDQ-Evidence compared to those databases.

This is the first study that has evaluated PDQ-Evidence. As far as we are aware, no other studies have been published that compare the use of different databases or search engines for finding health system evidence. Other studies have described the databases to which we compared PDQ-Evidence, but not specifically in relation to health system evidence, other than for Health Systems Evidence and EVIPNet VHL [4–6], both of which focus exclusively on health system evidence. Similarly, other studies have user tested other databases, for example, Cochrane Library [7], but not in comparison to another database and not with a focus on health system evidence.

## Conclusions

We conclude that PDQ-Evidence is at least as efficient as other databases and search engines for finding health system evidence and is probably more efficient. However, as with any search tool, there may be a learning curve and using PDQ-Evidence is not intuitive for some people. Moreover, preferences may vary. For example, some users value a focused database with limited content, such as PDQ-Evidence, whereas others prefer databases with a broader scope.

We believe that PDQ-Evidence is a useful tool for policy-makers, managers, their support staff, researchers and others with an interest in health system evidence. It has several unique features that help make it easy to use and easy to find a systematic review when one is available.

## Additional files

Additional file 1: PDQ-Evidence search strategies for PubMed, EMBASE, CINAHL, LILACS and PsycINFO. (DOCX 12 kb)

Additional file 2: Predefined and own questions. (DOCX 25 kb)

Additional file 3: Questionnaire: a comparative evaluation of PDQ-Evidence (PDQ first). (DOCX 24 kb)
