# Supplementary material for: A comparative evaluation of PDQ-Evidence
Source: Health Res Policy Syst. 2018 Mar 15;16:27. doi: 10.1186/s12961-018-0299-8 (PMC5856385; doi:10.1186/s12961-018-0299-8)
Supplement: Supplementary file 1 — PDQ-Evidence search strategies for PubMed, EMBASE, CINAHL, LILACS and PsycINFO. (DOCX 12 kb) [file 12961_2018_299_MOESM1_ESM.docx]

**Appendix 1. PDQ-Evidence search strategies for PubMed, EMBASE, CINAHL, LILACS, and PsycINFO**

**PubMed**

**Searched weekly**

#1. MEDLINE[Title/Abstract]

#2. (systematic[Title/Abstract] AND review[Title/Abstract])

#3. meta analysis[Publication Type]

#4. #1 OR #2 OR #3 (Methods filter for systematic reviews –Clinical Queries–Max Specificity)

#5. overview[Title] AND (reviews[Title] OR systematic[Title]

#6. meta-review[Title]

#7. review of reviews[Title]

#8. review[Title] AND systematic reviews[Title]

#9. umbrella[Title] AND (review[Title] OR reviews[Title] OR systematic[Title])

#10. policy[Title] AND (brief[Title] OR evidence[Title])

#11. #5 OR #6 OR #7 OR #8 OR #9 OR #10 (Methods filter for overviews)

#12. #4 OR #11 (Methods filter for systematic reviews and for overviews)

**EMBASE**

**Searched monthly**

Host: OVID

[meta-analysis.tw](http://meta-analysis.tw). OR systematic [review.tw](http://review.tw)

**CINAHL**

**Searched monthly**

Host: EBSCO

((TI meta analys* or AB meta analys*) or (TI systematic review or AB systematic review))

**LILACS**

**Searched weekly**

(TW:"revision sistematica" OR TW:"revisao sistematica" OR TW:"systematic review" OR MH:"review literature as topic" OR MH:"meta-analysis as topic" OR PT:"meta-analysis")

OR

(PT:revision AND (TW:metaanal$ OR TW:"meta-analysis" OR TW:"metaanalise" OR TW:"meta-analisis" OR TI:overview$ OR TW:"estudio sistematico" OR TW:"systematic study" OR TW:"estudo sistematico" OR TI:review OR TI:revisao OR TI:revision OR TI:systematic OR TI:sistematico))

OR

((TW:overview OR TW:"estudio sistematico" OR TW:"systematic study" OR TW:"estudo sistematico") AND (TI:review OR TI:revisao OR TI:revision OR TI:systematic OR TI:sistematico))

**PsycINFO**

**Searched monthly**

Host: EBSCO

meta-analysis OR search*
