# Supplementary material for: A comparative evaluation of PDQ-Evidence
Source: Health Res Policy Syst. 2018 Mar 15;16:27. doi: 10.1186/s12961-018-0299-8 (PMC5856385; doi:10.1186/s12961-018-0299-8)
Supplement: Supplementary file 2 — Predefined and own questions. (DOCX 25 kb) [file 12961_2018_299_MOESM2_ESM.docx]

# Appendix 2. Questionnaire: A comparative evaluation of PDQ-Evidence

# (PDQ first)

### **Welcome!** We invite you to help us evaluate various databases that include systematic reviews that address health system questions. Health system questions include questions about:

### Delivery arrangements (e.g. "The impact of specialist outreach clinics in primary care")

### Financial arrangements (e.g. "The impact of conditional cash transfers on use of health services")

### Governance arrangements (e.g. "How to manage dual practice among health workers")

### Implementation strategies (e.g. "How to improve antibiotic prescribing practices")

### Public health policies (e.g. "The impact of nutritional labelling for promoting healthier food consumption")

### We will ask you to:

### Give some background information about yourself

### Answer two health system questions, one predefined, and one of your own choosing, using three databases for each of the questions

### Rate the databases searched according to ease of use and time spent on searching

### For each of the two health system questions (the predefined one and the one of your own choosing) we will ask you to search for systematic reviews in three databases. These databases are:

### PDQ-Evidence, and two of the following ones (for you to choose):

### Cochrane Library

### EVIPNet VHL

### Google Scholar

### Health Systems Evidence

### PubMed

### Trip database

Please note that links to these databases need to be opened in a new tab or new window.

You should search PDQ-Evidence as the first database (**Database 1**), both for the predefined question and for the question of your own choosing.

It's up to you to decide the order in which the two databases that you will choose (**Database 2** and **Database 3**) should be searched for the same questions. 

Questions or fields marked with a red star are required.

You should use no more than 10 minutes per databases (30 minutes for each of the two questions).

You may save and later return to continue the evaluation where you left off.

## **Training or work experience**

## 1) Please mark any of the following areas where you have training or work experience

- Health policymaking
- Health systems planning or analysis
- Healthcare professional
- Management of health services
- Research
- Other - please specify below

## **Current position**

2) What type of position do you currently hold?

- Manager
- Policymaker
- Researcher
- Technical support staff or advisor
- Other - please specify below

## **First language**

#### 3) What is your first language?

- English
- French
- Norwegian
- Portuguese
- Spanish
- Other - please specify below

## **Searching**

#### 4) How often do you search for systematic reviews of health systems research?

- I never search myself
- Less than once a year
- Once or twice a year
- Once or twice a month
- Once or twice a week
- Many times a week

#### 5) Where do you normally search when you have a health systems question? Please list minimum 1 and maximum 3 sources (databases, web sites, etc.)

1:

2:

3:

Comments:

## **The predefined search question**

#### 6) Please select one of the following **predefined** health systems search questions

1. You are responsible for the community health services in your district. Due to the lack of health care personnel, you struggle to maintain the immunization program. Could community health workers step in and do some of the tasks that professionals normally would do?

2. Based on the just published “Gold standard for health care practice”, you realize that the health personnel you are responsible for are not in line with these standards. Do you think practice would improve if you discussed the results with the involved personnel after monitoring and giving them feedback on how they perform?

3. Staff at almost all maternal and child health centers in your region report that an increasing number of mothers opt out of the so far successful breast feeding program. Would some kind of financial incentives make these mothers change their mind?

4. The lack of primary care physicians in your district is chronic and critical. Could non-physicians do some of the work physicians normally do, and if so, who?

5. Having to pay for necessary health care might prevent poor people from using these services. Therefore, before introducing a fee-policy for health service, you wonder – will fees decrease the use of these fee-based services?

6. The Framework Convention on Tobacco Control proposes reducing the promotional impact of tobacco packaging. You are concerned about the high proportion of teenagers who smoke in your country and wonder if plain packaging would deter the onset of smoking by young people.

## **Your own search question**

### 7) Please write down a health systems search question **of your own choosing**

## **Choosing databases**

#### 8) Please select the **two databases** that you will use, in addition to PDQ-Evidence, to answer both the predefined question and the one of your own choosing

#### Please note that links to these databases need to be opened in a **new tab** or **new window**

- [Cochrane Library](http://www.thecochranelibrary.com/)
- [EVIPNet VHL](http://global.evipnet.org/)
- [Google Scholar](http://scholar.google.com/)
- [Health Systems Evidence](http://www.healthsystemsevidence.org/)
- [PubMed](http://www.ncbi.nlm.nih.gov/pubmed/)
- [Trip database](http://www.tripdatabase.com/)

## **Database 1 - PDQ-Evidence - predefined search question**

## 9) Please search [PDQ-Evidence](http://www.pdq-evidence.org/) to answer the predefined health systems search question (using no more than 10 minutes)

Please note that the link to PDQ-Evidence needs to be opened in a **new tab** or **new window**

#### 10) When did you start searching and when did you stop?

|  | **(e.g. Start 10:30 and Stop 10:37)** |
| --- | --- |
| **Start** |  |
| **Stop** |  |

#### 11) Did you find an answer?

- Yes
- No
- Comments

#### 12) If you found one or more systematic reviews that answer this question, please list up to three of the most relevant reviews that you found

|  | **First author** | **Title** | **Year of publication (4 digits)** |
| --- | --- | --- | --- |
| 1. |  |  |  |
| 2. |  |  |  |
| 3. |  |  |  |

## **Database 2 - own choice database - predefined search question**

### 13) This is the predefined health systems search question

#### 14) Please select and search one of the following two databases to answer the predefined health systems search question (using no more than 10 minutes)

Please note that links to these databases need to be opened in a **new tab** or **new window**

#### 15) When did you start searching and when did you stop?

|  | **(e.g. Start 10:30 and Stop 10:37)** |
| --- | --- |
| **Start** |  |
| **Stop** |  |

#### 16) Did you find an answer?

- Yes
- No
- Comments

#### 17) If you found one or more systematic reviews that answer this question, please list up to three of the most relevant reviews that you found

|  | **First author** | **Title** | **Year of publication (4 digits)** |
| --- | --- | --- | --- |
| 1. |  |  |  |
| 2. |  |  |  |
| 3. |  |  |  |

## **Database 3 - own choice database - predefined search question**

### 18) This is the predefined health systems search question

#### 19) Please select and search the following database to answer the predefined health systems search question (using no more than 10 minutes)

Please note that the link to this database needs to be opened in a **new tab** or **new window**

#### 20) When did you start searching and when did you stop?

|  | **(e.g. Start 10:30 and Stop 10:37)** |
| --- | --- |
| **Start** |  |
| **Stop** |  |

#### 21) Did you find an answer?

- Yes
- No
- Comments

22) If you found one or more systematic reviews that answer this question, please list up to three of the most relevant reviews that you found

|  | **First author** | **Title** | **Year of publication (4 digits)** |
| --- | --- | --- | --- |
| **1.** |  |  |  |
| **2.** |  |  |  |
| **3.** |  |  |  |

## **Database 1 - PDQ-Evidence - own search question**

### 23) Please search [PDQ-Evidence](http://www.pdq-evidence.org/) to answer your own health systems search question (using no more than 10 minutes)

Please note that the link to PDQ-Evidence needs to be opened in a **new tab** or **new window**

#### 24) When did you start searching and when did you stop?

|  | **(e.g. Start 10:30 and Stop 10:37)** |
| --- | --- |
| **Start** |  |
| **Stop** |  |

#### 25) Did you find an answer?

- Yes
- No
- Comments

#### 26) If you found one or more systematic reviews that answer this question, please list up to three of the most relevant reviews that you found

|  | **First author** | **Title** | **Year of publication (4 digits)** |
| --- | --- | --- | --- |
| **1.** |  |  |  |
| **2.** |  |  |  |
| **3.** |  |  |  |

## **Database 2 - own choice database - own search question**

### 27) This is your own health systems search question

#### 28) Please select and search one of the following two databases to answer your own health systems search question (using no more than 10 minutes)

Please note that links to these databases need to be opened in a **new tab** or **new window**

#### 29) When did you start searching and when did you stop?

|  | **(e.g. Start 10:30 and Stop 10:37)** |
| --- | --- |
| **Start** |  |
| **Stop** |  |

#### 30) Did you find an answer?

- Yes
- No
- Comments

#### 31) If you found one or more systematic reviews that answer this question, please list up to three of the most relevant reviews that you found

|  | **First author** | **Title** | **Year of publication (4 digits)** |
| --- | --- | --- | --- |
| **1.** |  |  |  |
| **2.** |  |  |  |
| **3.** |  |  |  |

## **Database 3 - own choice database - own search question**

### 32) This is your own health systems search question

#### 33) Please select and search one of the following two databases to answer your own health systems search question (using no more than 10 minutes)

Please note that links to these databases need to be opened in a**new tab**or**new window**

#### 34) When did you start searching and when did you stop?

|  | **(e.g. Start 10:30 and Stop 10:37)** |
| --- | --- |
| **Start** |  |
| **Stop** |  |

#### 35) Did you find an answer?

- Yes
- No
- Comments

#### 36) If you found one or more systematic reviews that answer this question, please list up to three of the most relevant reviews that you found

|  | **First author** | **Title** | **Year of publication (4 digits)** |
| --- | --- | --- | --- |
| **1.** |  |  |  |
| **2.** |  |  |  |
| **3.** |  |  |  |

## **Satisfaction with databases searched - ease of use**

#### Please rank the databases you have searched according to ease of use

|  | **Very difficult** | **Difficult** | **Easy** | **Very easy** |
| --- | --- | --- | --- | --- |
| **PDQ-Evidence** |  |  |  |  |
|  |  |  |  |  |
|  |  |  |  |  |

## **Satisfaction with databases searched - time spent**

#### Please rank the databases you have searched according to time spent

|  | **Much too much time** | **Too much time** | **Not too much time** | **Very little time** |
| --- | --- | --- | --- | --- |
| **PDQ-Evidence** |  |  |  |  |
|  |  |  |  |  |
|  |  |  |  |  |

## **Comments to PDQ-Evidence**

#### 37) For PDQ-Evidence, please comment on the following

|  | **PDQ-Evidence** |
| --- | --- |
| **What you liked** |  |
| **What you disliked** |  |
| **Challenges** |  |
| **Suggestions for improvements** |  |

### **Thank you for taking our survey.**
