# Supplementary material for: A comparative evaluation of PDQ-Evidence
Source: Health Res Policy Syst. 2018 Mar 15;16:27. doi: 10.1186/s12961-018-0299-8 (PMC5856385; doi:10.1186/s12961-018-0299-8)
Supplement: Supplementary file 3 — Questionnaire: a comparative evaluation of PDQ-Evidence (PDQ first). (DOCX 24 kb) [file 12961_2018_299_MOESM3_ESM.docx]

| **Predefined questions** | |
| --- | --- |
|  | You are responsible for the community health services in your district. Due to the lack of health care personnel, you struggle to maintain the immunization program. Could community health workers step in and do some of the tasks that professionals normally would do? |
|  | Based on the just published “Gold standard for health care practice”, you realize that the health personnel you are responsible for are not in line with these standards. Do you think practice would improve if you discussed the results with the involved personnel after monitoring and giving them feedback on how they perform? |
|  | Staff at almost all maternal and child health centers in your region report that an increasing number of mothers opt out of the so far successful breast feeding program. Would some kind of financial incentives make these mothers change their mind? |
|  | The lack of primary care physicians in your district is chronic and critical. Could non-physicians do some of the work physicians normally do, and if so, who? |
|  | Having to pay for necessary health care might prevent poor people from using these services. Therefore, before introducing a fee-policy for health service, you wonder – will fees decrease the use of these fee-based services? |
|  | The Framework Convention on Tobacco Control proposes reducing the promotional impact of tobacco packaging. You are concerned about the high proportion of teenagers who smoke in your country and wonder if plain packaging would deter the onset of smoking by young people. |

| **Own questions** | | | |
| --- | --- | --- | --- |
|  | Is there benefit of aspirin in primary prevention |  | What interventions would increase adherence to clinical guidelines by healthcare workers? |
|  | Does telepsychiatry provide a viable alternative to face to face consultations in low resource settings? |  | Could mobile phone text messaging improve adherence to anti-tuberculosis treatment? |
|  | Does pay for performance change the behaviour of primary health professionals? |  | what is the effects of observation units in hospitals? |
|  | What is the impact of task shifting for HIV-positive patients? |  | What is the impact of western diet and sedentary lifestyle in childhood obesity in Africa? |
|  | Do fee for service payment mechanism improve medical productivity compared with salary payment? |  | You are part of the pharmaceutical cost containment team of the Ministry of Health from your country. Concerned about the increased expenditure of the last years, which will be the best intervention, the best policy to apply ? |
|  | Do healthcare professionals manage their waste appropriately in hospital settings? |  | What community based interventions can improve early detection of breast cancer in developing countries |
|  | financial incentive to improve utilization of facility childbirths in lo-and-middle-income countries |  | How do you increase the uptake of family planning services in a crisis setting? |
|  | How can health systems in developing countries provide effective disease prevention and treatment to all. |  | Hospitals are struggling to improve their patient satisfaction scores. Would post discharge phone call to the patient by the physician improve that score? |
|  | What is the effect of pricing/taxation on non-cigarette tobacco products? |  | Does the use of community healthcare advisers improve adherence of ARV medicines? |
|  | As with the uprising levels of nosocomial infections, regardless of the hospitals precautionary efforts, what new measures would help to control the rates of nosocomial infections? |  | Does a pay for performance scheme improve performance in primary care? |
|  | Will training of traditional birth attendants improve uptake of HCT by mothers? |  | What is the most effective strategy for implementing national guidelines? |
|  | What interventions have worked to prevent or control corruption in health systems? |  | Can midwives working in antenatal clinics in community settings in LMICs, safely and effectively use ultrasound to assess gestational age in low-risk women at <20 weeks gestation |
|  | In residency training, which model is better to achieve best patient care during out-patient rotations; the 4+1 model or the half-day clinic per week? |  | Recently, there has been a rise in incidence of hypertension related strokes attributable to missed clinic visits. Could setting up facility based phone SMS reminders improve clinic attendance and reduce stoke incidence? |
|  | In addition to individual approaches, eco-environmental strategies related to the availability of products have shown positive effects in changing lifestyle behaviors. You are concerned about the high proportion of obesity and the related eating behavior among young people in your region and wonder if the availability of healthy food in different sites of your region would change the eating behavior of the youth and decrease the proportion of obesity among them |  | Is screening for colon cancer beneficial for general population |
|  | The effectiveness of Group Clinics for Chronic Disease |  | Could community health workers do some of the tasks that professionals normally would do? |
|  | Nutritional interventions that reduce overweight rate or obesity among pre-school children |  | What is the impact of training community health workers to refer household members to health clinics? |
|  | Does the use of electronic health records in the exam room affect patient-physician relationship? |  | How to improve antibiotic prescription practices |
|  | Does free-fees based service policy a good strategy to render healthcare services to disadvantage communities? |  | Giving U500 insulin after stopping the insulin drip prevents blood glucose spiking up if patient doesn't start the pump after surgery? |
|  | What is the best way of providing oral health care to elderly people in long-term residential care? |  | are family health teams more cost-effective than solo practice fee for service general practitioners |
|  | What is the effect of interventions designed to improve the transition of care for adolescents from paediatric to adult health services |  | The lack of primary care physicians is not improving. Could nurse practitioners do some of the work physicians normally do? |
|  | Poverty is associated with increased risks of acquisition of infectious diseases in low and middle income countries. Would improved socioeconomic interventions in these settings reduce the risks of acquisition of these infections? |  | How to impove a health benefit package? |
|  | Does micro-nutrient supplementation during pregnancy improve growth outcomes of children in low income countries? |  | community health workers OR lay health workers OR task-shifting AND immunization OR immunisation OR vaccination |
|  | Interventions to improve antenatal care coverage |  | How effective are decentralized services for the management of non-communicable diseases. |
|  | Gynecologists are not attached to newly distributed national guideline for Cesarean Section indications. Will financial incentives promote them to follow the new guideline? |  | Impact of public private partnership in improving the health status of the population |
|  | Will implementing shorter/normal working hours for doctors lead to fewer medical errors in patients? |  | How large capacity do services for child mental health have in western countries? |
|  | Does setting a fixed fee for medicines at manufacturer level reduce medicine prices and increase access to medicines? |  | Do parent training interventions improve communication skills in autisic children |
|  | Does giving personalized feedback to clinicians improve guideline adherence. |  | What are the benefits of group prenatal care above and beyond individually delivered care? |
|  | Medicines for chronic CVD diseases including diabetes might be used insufficiently in health system due to several factors. What are the underlying causes of diabetes medicines under utilization? |  | In Brazil many professional strongly argue about the importance of pharmacists to participate on the health care process, mainly at primary health care level. What are the most important set of tasks trough which pharmacist can contribute to positive health outcomes? |
|  | community acceptability of HPV vaccination for boys |  | ACT-team for outreach of mental health services |
|  | Can community health workers/volunteers complete some of the screening tasks that are usually done by health professionals? |  | How often should people be invited in for a routine dental check-up? |
|  | Does Integration of primary health care improve the health status of population in developping countries |  | In order to achieve universal health coverage, various health financing models are being considered. What could be the most appropriate financing model to cover informal sector populations? |
|  | In task shifting, does differences in compensation between non-physicians and physicians discourage service delivery |  | is the increase of physical activity at school effectyive in reducing incidence of obesity and anorexia in kids? |
|  | Are handheld devices (tablets, phones) effective implementation tools for clinical practice guidelines? |  | Is Positron Emission Tomography (any tracer) recommended to be provided by the public health insurance system in order to assist the diagnosis of Alzheimer's disease within an available program for the management of these patients? |
|  | Would plain packaging deter the onset of smoking by young people. |  | impact of regionalisation of surgical procedures |
|  | You want to know if it is possible to improve compliance to the guidelines for best treatment with antibiotics. How health operators non-physician may be involved in this? |  | Is non-medical staffs' involvement to primary prevention of metabolic syndrome really cost-effective? If so, how to train them? |
|  | what would bring to vaccinate the boys against HPV |  | Could mobile phones be used to effectively help in improving reporting rates for births and deaths in the community? |
|  | What is the effect of Humira on remission in Crohns disease? |  | Do health professionals in hospitals embrace clinical leadership and if so how does this influence performance |
|  | Immunization strategies for polio in rural Nigeria |  | Non communicable diseases are on the rise especially in SubSaharan Africa; what plans does the government have to purchase health care services for the poorest? |
|  | What is the impact of nutritional labeling on promoting healthier food consumption? |  | Can imposing minimal number of interventions for certain procedures provide wrong incentives to clinicians to perform these interventions in inappropriate indications? |
|  | The effectiveness of mHealth to improve access to skilled birth attendants during labour, delivery and 24 hours post-partum |  | Dose promoting exercise during pregnancy improve health in newborn and mothers? |
|  | will follow-up by telephone from the GP increase compliance of drugs prescribed compared to no telephone follow-up? |  | Can I improve early diagnosis of coeliac disease by offering point of care testing? |
|  | would routine prostate screening with PSA reduce mortality from prostate cancer? |  |  |
